# Finding exclusively deleted or amplified genomic areas in lung adenocarcinomas using a novel chromosomal pattern analysis

**DOI:** 10.1186/1755-8794-2-43

**Published:** 2009-07-14

**Authors:** Philippe Broët, Patrick Tan, Marco Alifano, Sophie Camilleri-Broët, Sylvia Richardson

**Affiliations:** 1 Computational & Mathematical Biology, Genome Institute of Singapore, Singapore, Republic of Singapore; 2 JE2492, Faculty of Medicine Paris-Sud, Bicêtre, France; 3 Department of thoracic surgery, Assistance Publique-Hôpitaux de Paris, Paris, France; 4 Cancer & Stem Cell Biology, Duke-NUS Graduate Medical School, Republic of Singapore; 5 Centre for Biostatistics, Imperial College London, Norfolk Place, London, W2 1PG, UK

## Abstract

**Background:**

Genomic copy number alteration (CNA) that are recurrent across multiple samples often harbor critical genes that can drive either the initiation or the progression of cancer disease. Up to now, most researchers investigating recurrent CNAs consider separately the marginal frequencies for copy gain or loss and select the areas of interest based on arbitrary cut-off thresholds of these frequencies. In practice, these analyses ignore the interdependencies between the propensity of being deleted or amplified for a clone. In this context, a joint analysis of the copy number changes across tumor samples may bring new insights about patterns of recurrent CNAs.

**Methods:**

We propose to identify patterns of recurrent CNAs across tumor samples from high-resolution comparative genomic hybridization microarrays. Clustering is achieved by modeling the copy number state (loss, no-change, gain) as a multinomial distribution with probabilities parameterized through a latent class model leading to nine patterns of recurrent CNAs. This model gives us a powerful tool to identify clones with contrasting propensity of being deleted or amplified across tumor samples. We applied this model to a homogeneous series of 65 lung adenocarcinomas.

**Results:**

Our latent class model analysis identified interesting patterns of chromosomal aberrations. Our results showed that about thirty percent of the genomic clones were classified either as "exclusively" deleted or amplified recurrent CNAs and could be considered as non random chromosomal events. Most of the known oncogenes or tumor suppressor genes associated with lung adenocarcinoma were located within these areas. We also describe genomic areas of potential interest and show that an increase of the frequency of amplification in these particular areas is significantly associated with poorer survival.

**Conclusion:**

Analyzing jointly deletions and amplifications through our latent class model analysis allows highlighting specific genomic areas with exclusively amplified or deleted recurrent CNAs which are good candidate for harboring oncogenes or tumor suppressor genes.

## Background

Chromosomal instability plays an important role in carcinogenesis with numerical and structural genomic alteration leading to selective growth advantages [1]. In recent years, high-resolution array comparative genomic hybridization (aCGH) has replaced conventional metaphase CGH as the standard protocol for identifying segmental copy number alteration across the whole genome. The classical strategy of aCGH technique is to co-hybridize genomic DNA from a cancer sample (labelled with one fluorochrome) with genomic DNA from a normal reference sample (labelled with a different fluorochrome) to the aCGH targets. These targets correspond to chosen genomic clones or non-overlapping oligonucleotides of different lengths that are spotted or directly synthesized onto the solid support. In practice, the distribution and length of the spotted array elements determine the detection sensitivity to various alteration sizes with some recent platforms being able to detect alteration sizes less that 100-kb [2].

In clinical cancer research, large collections of tumor samples are currently being analyzed using aCGH experiments. After assessing regions with copy gains or losses within each individual sample, the main challenge is to identify genomic areas where amplifications or deletions are recurrent across tumor samples and hypothesized to harbour oncogenes or tumor suppressor genes of interest. More precisely, the challenge is to distinguish between "bystander" and "driver" chromosomal aberrations, these latter changes conferring biological properties to the tumor that allow it to proliferate.

In order to identify these functionally and potentially clinically important chromosomal changes, classical approaches focus on loss and gain as separate cases and select aberrations that are deemed significant using ad-hoc frequency thresholds or permutation-based method [3-5]. A shortcoming of these methods is that they analyze copy loss and copy gain as separate events without considering jointly the chromosomal propensity for deletions and amplifications. However, genomic areas harboring either oncogenes or tumor suppressor genes should jointly exhibit high frequency amplification together with a low frequency deletion, and vice versa, respectively. Thus, the ability to identify these "driver" chromosomal aberrations should be improved by modeling jointly the occurrence of deletions and amplifications across the tumor samples.

To achieve this, we propose a novel strategy to identify patterns of recurrent copy number alteration (CNA) based on a latent class model framework. Here, a pattern is considered to be a model-based representation of a clone's propensity for exhibiting chromosomal aberrations (deletion and amplification) in a specific disease entity. Based on these patterns, we highlight genomic areas having the highest frequency for amplification together with the lowest frequency for deletion (so called *exclusively *amplified CNA) and vice versa (so called *exclusively *deleted CNA). A case study that investigated CNAs in a homogeneous series of sixty-five early stage lung adenocarcinomas using 32K BAC arrays is analyzed to demonstrate the interest of this approach. In particular, we identified regions exhibiting a high rate of amplification together with a low rate of deletion that are likely to confer a selective advantage and probably harbor one or several oncogenes. We also analyse the potential impact of an accumulation of such chromosomal aberrations on patients' outcomes.

## Methods

### Data and preprocessing

The dataset considered in this study is based on a homogeneous series of 65 patients with stage IB lung adenocarcinomas (excluding large cell carcinomas) who underwent surgery (AP-HP, France). This study was approved by the Hôtel-Dieu hospital ethic committee. DNA was extracted from frozen sections using the Nucleon DNA extraction kit (BACC2, Amersham Biosciences, Buckinghamshire, UK), according to the manufacturer's procedures. For each tumor, two micrograms of tumor and reference genomic DNAs were directly labeled with Cy3-dCTP or Cy5-dCTP respectively and hybridized onto aCGH containing 32,000 DOP-PCR amplified overlapping BAC genomic clones (average size of 200 kb) providing tiling coverage of the human genome. Hybridizations were performed using a MAUI hybridization station, and after washing, the slides were scanned on a GenePix 4000B scanner. For this analysis, we only considered BAC genomic clones mapping to automosomal chromosomes. The aCGH signal intensities were normalized using a two-channel microarray normalization procedure. For each sample, inferences about the copy number status of each BAC clone were obtained using the CGHmix classification procedure [6]. In practice, we compute the posterior probabilities of a clone belonging to either one of the three defined genomic states (loss, modal/unaltered and gain copy state) from a spatial mixture model framework. Then, we assigned each clone to one of two modified copy-number allocation states (loss or gain copy state) if its corresponding posterior probability was above a defined threshold value, otherwise the clone was assigned to the modal/unaltered copy state. This latter threshold value was selected to obtain the same false discovery rate of 5% for each sample. Here, a false discovery corresponded to a clone incorrectly defined as amplified or deleted by our allocation rule.

### Model

Let  denote the 3-dimensional random variable which records the number of deletions , amplifications  and modal copy  observed for genomic clone *i *(*i *= 1, ..., *I*) over the sample set of tumors with size *n*. Let *L*_*i *_be an unobserved (latent) categorical allocation variable taking the values 1, ..., K with probabilities *w*_1_, ..., *w*_*K*_, respectively. Here, *L*_*i *_indicates the index of the class to which genomic clone *i *belongs. These classes are a convenient representation for describing CNA patterns in term of their propensity for amplification and deletion. The class variable is not observed and hence said to be latent. As seen below, we consider a latent class model with three levels (low, medium, high)for both amplification (*j *= 1,2,3) and deletion (j* = 1,2,3) leading to nine latent classes (*K *= 9).

For a genomic clone *i *belonging to class *k *= (*j*, j*), we assume that *Y*_*i *_follows a multinomial distribution (here a trinomial distribution) with conditional response probabilities for loss copy state (deletion) , gain copy state (amplification)  and modal copy state  parameterized with the latent class parameters  (deletion) and  (amplification) such as:



Given these probabilities, we define the conditional distribution of *Y*_*i *_as:



Or equivalently



Thus, we have implicitly assumed that any dependence of copy number anomalies between clones is captured by the latent class structure. It follows that the marginal cumulative distribution function of *Y*_*i *_comes from a mixture model:



where the quantities *w*_*k *_Pr (*L*_*i *_= *k*) are the mixing proportions or weights with 0 ≤ *w*_*k *_≤ 1 and . For identifiability, we impose that  and .

We summarize the labelling of the nine latent classes in Table 1 and retain the double indexing *k *= (*j*, j*)when needed for ease of understanding.

**Table 1:** Labeling of the nine latent classes

	Low	Medium	High
Low	k = 1;(α_j = 1_^A^;α_j* = 1_^D^)	k = 2;(α_j = 1_^A^;α_j* = 2_^D^)	k = 3;(α_j = 1_^A^;α_j* = 3_^D^)

Medium	k = 4;(α_j = 2_^A^;α_j* = 1_^D^)	k = 5;(α_j = 2_^A^;α_j* = 2_^D^)	k = 6;(α_j = 2_^A^;α_j* = 3_^D^)

High	k = 7;(α_j = 3_^A^;α_j* = 1_^D^)	k = 8;(α_j = 3_^A^;α_j* = 2_^D^)	k = 9;(α_j = 3_^A^;α_j* = 3_^D^)

### Inference

For each latent class *k *= (*j*, j*), our purpose is to estimate the parameters  and  together with the posterior probability of belonging to one of the *K *classes for each genomic clone *i*. We consider a Bayesian framework, where ,  and *w*_*k *_are given prior distributions. Here, the prior distributions specify that these quantities are all drawn independently, with Normal ( and ) and Dirichlet priors (*w*_*k*_). In practice,  and  are given independent normal prior distributions with large variance. The parameter δ of the symmetric prior Dirichlet distribution was set to 0.5 (Jeffreys' prior), instead of the usual value of 1 that corresponds to uniform weights, in order to be less informative.

Inference for parameters of interest was undertaken by sampling from their joint posterior distributions using Monte Carlo Markov chain (MCMC) samplers implemented in the WinBUGS software [7]. All results presented correspond to 5,000 sweeps of MCMC algorithms following a burn-in period of 1,000 (period for achieving stability of the algorithm). Summary statistics for quantities of interest, such as  and  were calculated from the full output of the MCMC algorithm. Furthermore, the samples provided information on quantities of prime interest, the vector of the posterior probabilities for each genomic clone *i *of belonging to class *k*: *p*_*i *_= {pr(*L*_*i *_= *k *| *data*); *k *= 1, ..., 9} These posterior probabilities are directly estimated as empirical averages from the output of the algorithm. Using these estimates, a probabilistic clustering of the data can be achieved. To be specific, we chose to apply the Bayes classification rule and assigned each clone to the class to which it had the highest probability of belonging. We stress that the classes capture chromosomal aberration patterns.

In this work, we compared seven different latent class models with various levels of amplification and deletion (corresponding to 2, 3 and 4 levels of copy gain and copy loss). For each model, we computed the Deviance Information Criterion (DIC) as introduced by Spiegelhalter et al. [8] and extended for mixture models as proposed by Richardson [9]. Models with small DIC provide a better fit than those with high DIC criteria. Thus the number of latent levels can be adapted to the particular cancer investigated and the observed chromosomal patterns in the sample.

## Results

### Chromosomal pattern analysis

In our dataset, several competing models were tenable ranging from six to nine components. We heuristically chose to favor the nine-component model which leads to a good fit and allow a sufficient number of components for describing finely the different levels of genomic aberrations across the whole dataset.

**Figure 1:**
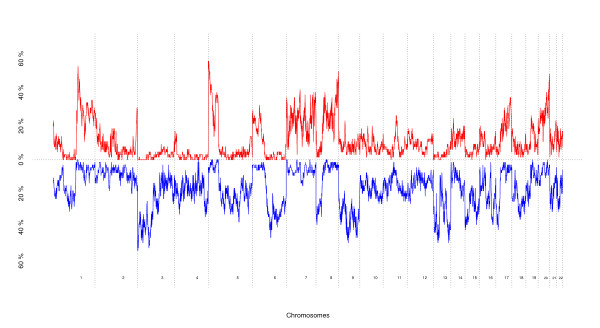
**Frequencies of chromosomal aberrations**. The frequencies of amplification (red) and deletion (blue) over the 65 lung adenocarcinomas are plotted and ordered, according to the chromosomal order (x-axis) from 1 pter to 22 qter.

Figure 1 displays the frequencies of amplification (red) and deletion (blue) of 29,691 BACs located on autosomal chromosomes over the 65 lung adenocarcinomas according to the chromosomal order from 1 pter to 22 qter. These results are consistent with previous reports investigating losses and gains in lung adenocarcinomas [10-12], supporting a complex mesh of copy number alterations in lung carcinogenesis.

**Figure 2:**
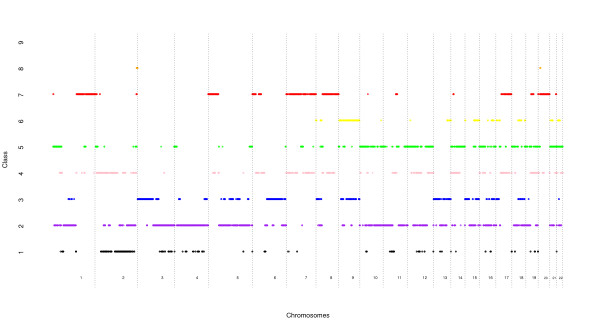
**Chromosomal aberration patterns**. The allocation of the 29,691 BC clones (in one of the nine classes) obtained from our latent class model analysis and considering a Bayes classification rule. Exclusively amplified recurrent CNAs are in class k = 7 (red) whereas exclusively deleted recurrent CNAs are in class k = 3 (blue).

Probabilistic clustering of the BACs obtained from our latent class model analysis is shown in Figure 2. We observed a mixture of broad and focal contiguous genomic areas with the same patterns of CNAs.

Tables 2 displays for the nine classes the joint estimated average probabilities for amplification and deletion, respectively. Probability for amplification ranges from 3.0% to 29.7% whereas for deletion it ranges from 5.4% to 34.5%. Note that arbitrary probability cut-offs were not imposed to define the classes, rather the observed propensities were flexibly clustered through the latent class model. Table 3 summarizes the number of clones allocated in each class (and corresponding percentage) applying the Bayes classification rule. The class with the highest levels for deletion and amplification (*k *= 9) is empty. The class with medium rate of deletion and low rate of amplification (*k *= 2) regrouped the highest number of clones (9,509).

**Table 2:** Joint estimated average probabilities of amplification/deletion for the nine classes

	Low	Medium	High
Low	4.3%; 7.3%	3.7%; 18.6%	3.0%; 34.5%

Medium	13.6%; 6.7%	12.1%; 17.0%	9.9%; 32.0%

High	29.7%; 5.4%	27.0%; 14.1%	22.8%; 27.4%

The two percentages given in each cell represent the frequency of amplification and deletion, respectively

**Table 3:** Number (proportion) of genomic clones for the nine classes applying the Bayes classification rule (assign each clone to the class to which it had the highest probability of belonging)

	Low	Medium	High
Low	1,567 (5.3%)	9,509 (32.0%)	4,481 (15.1%)

Medium	3,426 (11.5%)	4,497 (15.2%)	1,283 (4.3%)

High	4,854 (16.3%)	74 (0.2%)	0 (0.0%)

Some interesting patterns emerge from Tables 2, 3 and Figure 2. From a biological point of view, four sets of genomic clones have patterns that are particularly worth highlighting.

The first set is composed of clones from class *k *= 1, that exhibit simultaneously very low deletion and amplification rates. This group may be interpreted as "refractory" clones with aberration rate below chromosomal background (corresponding to random chromosomal aberrations as defined below). As seen from our results, this set is small gathering only 5.3% of the total number of clones. The second set is composed of clones from classes *k *= 2, 4 and 5 with medium values of either deletion or amplification rates that can be considered as chromosomal background rate of aberrations. This set gathers about two-third of the total number of clones and may be interpreted as regrouping clones with random chromosomal aberrations.

The third and most interesting set is composed of approximately 9,000 clones from classes *k *= 3 and *k *= 7 with very high rate for either deletion or amplification associated with refractory status (below the chromosomal background rate of aberration) for the converse copy state. We refer to the clones in class *k *= 7 as "exclusively amplified" recurrent CNAs and those in class *k *= 3 as "exclusively deleted" recurrent CNAs. It can be hypothesized that these "exclusive" behaviors reflect a selective advantage for tumor growth for one state (e.g. amplification) associated with a selective disadvantage of the converse state (e.g. deletion). Thus, it is likely that this set contains "driver" clones, harboring functionally important changes giving selective advantage to tumor cells.

The last set is composed of clones belonging to class *k *= 6 and *k *= 8 that exhibit a complex pattern with high and medium values for both amplification and deletion. These classes may be interpreted as regrouping genomic regions that contain multiple genes that contribute to cancer, some of which being selected for copy gain and other for copy loss. In particular, we identified genomic clones located within cytogenetic band 16q23 that are classified in class *k *= 6 and harbor both the tumor suppressor gene WWOX and the oncogene MAF.

Modeling jointly the occurrence of amplifications and deletions across the tumor samples allows us to identify such patterns. To assess the biological relevance of the patterns found, we examined whether known lung cancer genes were classified as "exclusively amplified" or "exclusively deleted" recurrent CNAs. We found that, with exception of PTEN, all the oncogenes and tumor suppressor genes known to be associated with quantitative genomic changes in lung adenocarcinoma [10-12] were classified as "exclusively amplified" (*k *= 7) or "exclusively deleted" (*k *= 3) recurrent CNAs (Table 4). It is worth noting that PIK3CA gene (3q26.3 locus), described as specifically amplified in another histological subtype (squamous lung carcinomas) [9], was not found within an "exclusively" recurrent CNA emphasizing the histological homogeneity of our series and the specificity of the "exclusively" amplified or deleted classes.

**Table 4:** Oncogenes and tumor suppressor genes known to be associated with genomic changes in lung adenocarcinoma

**Gene**	**CNA class**	**Cytoband**	**Deletion (%)**	**Amplification (%)**
FHIT	k = 3 (D)	3p14.2	52.3	1.5

LIMD1	k = 3 (D)	3p21.3	34.4	1.5

hTERT	k = 7 (A)	5p15.33	6.2	55.4

SKP2	k = 7 (A)	5p13	1.5	38.5

EGFR-1	k = 7 (A)	7p11.2	1.5	24.6

c-MET	k = 7 (A)	7q31	7.7	23.1

c-MYC	k = 7 (A)	8q24.12-13	4.6	36.9

CDKN2A	k = 3 (D)	9p21	29.2	6.2

PTEN	k = 2 (D)	10q23.3	18.5	5.2

CCND1	k = 7 (A)	11q13	6.2	21.5

RB	k = 3 (D)	13q14.2	43.1	1.5

NKX2-1	k = 7 (A)	14q13.3	3.1	21.5

WWOX	k = 3 (D)	16q23.3-24.1	33.8	6.2

P53	k = 3 (D)	17p13.1	36.9	4.6

E2F	k = 7 (A)	20q11.2	3.1	23.1

**Figure 3:**
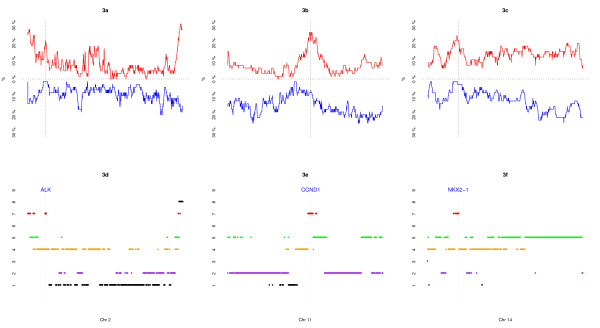
**Chromosomal patterns for chromosome 2, 11, 14**. The frequencies of amplification and deletion over the 65 lung adenocarcinomas detailed for chromosomes 2, 11 and 14 (3a, 3b, 3c). Group allocation of BAC clones for chromosome 2, 11 and 14 (3d, 3e, 3f). with locations of known oncogenes and tumor-supressor genes (ALK, CCND1, NKX2-1).

In Figure 3, we look in greater detail at three selected chromosomes (Chromosome 2, 11 and 14) harboring genomic areas classified as "exclusively amplified" recurrent CNAs.

In chromosome 2, we identified a focal area located within the 2p23 locus which harbors the ALK oncogene (anaplastic lymphoma receptor tyrosine kinase). This gene which is known to play a role in lymphomas has been recently shown to be activated in lung cancer either by gene fusion with EML4 or amplification [13,14].

In chromosome 11, we identified a short area located within the locus 11q13.2 which harbors the well-known oncogene CCND1. In a validation analysis, we analyzed protein expression by immunohistochemistry and found that CCND1 amplification was significantly related with gene over-expression (data not shown). We also identified a second small genomic area with "exclusively amplified" recurrent CNAs located within the locus 11q13.4-13.5. This area contains several candidate genes including the Neu3 gene (Human plasma membrane-associated sialidase) which is upregulated in several human cancers and is known to interact with EGFR. Except for these loci, most of the chromosome harbors clones from class *k *= 2 with medium values of deletion rates and low level of amplification that can be considered with random chromosomal aberrations.

In chromosome 14, we identified the recently described focal area of amplification located within the 14q13.3 locus which harbors the NKX2-1 gene [11]. This gene encodes for the well known TTF1 (Thyroid transcription factor), a protein which is expressed in normal lung and thyroid tissues and in their related adenocarcinomas. Showing NKX2-1 gene located within an "exclusively amplified" recurrent CNAs favors the hypothesis that TTF1 gene product may have a functional role in lung carcinogenesis instead of just being a marker of primary lung origin.

We then compare our results to those obtained from previously used methods that consider arbitrary thresholding rules (frequency cutoffs of 20%, 25% and 30%) or permutation-based approaches. As seen in Table 4, an arbitrary threshold of 20% leads to the selection of the known oncogenes/tumor suppressor genes whereas the widely used 25% threshold will discard interesting genes such as EGFR-1, c-MET, CCND1, NKX2-1 and E2F. However, the 20% threshold selects a high proportion of the genome (50.5% of the total number of clones) whereas our method selects only 31.4% (9,335 clones) which is comparable to the 25% thresholds (33.6% of the total number of clones).

We also analyzed our data using the method proposed by Klijn et al. [4] that has been previously shown to outperform the one proposed by Diskin et al. [3]. The Klijn et al. method (called KC-SMART) is implemented in the R/Bioconductor package [15] and the null hypothesis is obtained by shuffling the non-discretized data (log-ratio data) over the entire genome. Considering a false discovery rate level of 5% seems inappropriate since it leads to select too many genomic areas (>80%). For a family wise error rate of 5% (with a 4 Mb kernel width), we selected 3,663 (12.3%) recurrent deletions and 2,524 (8.5%) recurrent amplification. Forty nine percent of these recurrent amplifications are classified by our approach as "exclusively amplified" recurrent CNAs, the others belonging to classes with medium amplification rate. We observe that the KC-SMART selection of amplified areas ignores important genomic areas that we classified as "exclusively amplified" such as those harboring MET gene. Moreover, no genomic area belonging to class 8 was selected even when considering various kernel widths. This is not surprising since null hypotheses for detecting marginally amplification or deletion are highly dependent on the definition of the "complementary" state (e.g. for deletion the "complementary" state corresponds to modal or gain copy). For the 3,663 selected recurrent deletions by KC-SMART, 34.7% and 30.7% are classified by our approach in class 3 and 2 respectively whereas the other clones belong to classes with medium deletion rate. This selection does not recognize some genomic regions that we classified as "exclusively deleted" such as those harboring WWOX tumor suppressor gene. As could be expected, this procedure selects a subset of amplified (respectively deleted) clones that have a variety of deletion (respectively amplification) rates, whereas our modeling approach is aimed at refining this characterization, by focusing on highlighting clones with contrasting patterns of amplification and deletion.

### Relationship between chromosomal patterns and clinical outcome

Finally, we analyzed the impact of chromosomal aberrations on relapse-free survival (gain and loss considered separately since they have distinct impact on the disease) calculated from the date of the patients' surgery until either disease related death, disease recurrence or last follow-up examination. More specifically, we investigated whether chromosomal pattern information obtained by our latent class model could be useful for distinguishing genomic regions prone to non-random chromosomal event (signal) and with potential impact on clinical outcome from those prone to random chromosomal event (noise).

In practice and for copy gain, we calculate for each patient two different scores that measure the proportion of copy gains over the selected genomic regions. The first score is computed over the 4,4854 genomic clones prone to non-random chromosomal event that belong to "*exclusively *amplified regions" (class *k *= 7 as defined previously). The second score is computed over 17,432 genomic clones prone to random chromosomal event (classes *k *= 2, *k *= 4 and *k *= 5).

**Figure 4:**
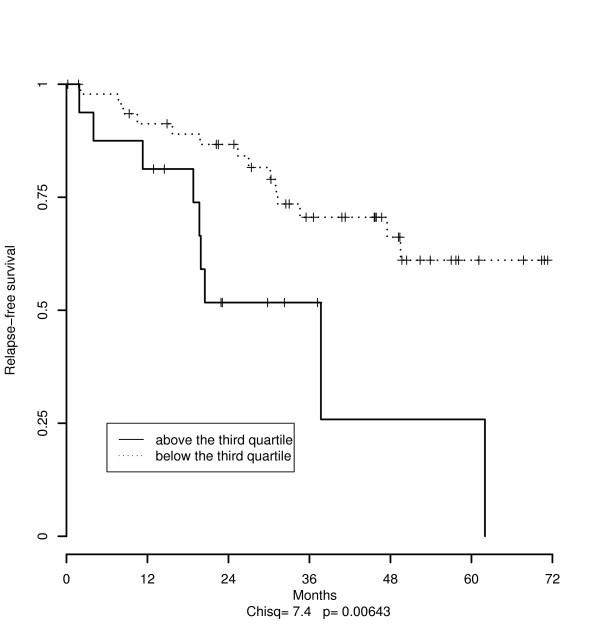
**Relapse-free survival from high and low-risk groups according to the proportion of chromosomal amplifications**. RFS curves for the 65 lung adenocarcinomas considering high and low-risk groups. High-risk group are those with a high proportion of amplification (above the third quartile, plain line) whereas low-risk group are those with low proportion of amplification (above the third quartile, dash line). These proportions are computed over genomic clones prone to non-random chromosomal event that belong to "exclusively amplified regions" (class 7 as defined in our model).

The median value of the scores measured over genomic clones from class *k *= 7 was of 28.8% [first quartile = 16.1, third quartile = 42.4] whereas it was of 23.6% [first quartile = 11.7, third quartile = 34.2] for genomic clones from classes *k *= 2, *k *= 4 and *k *= 5. The results from the Cox proportional hazard regression model, considering each score as a continuous variable, showed that an increasing proportion of copy gains within "*exclusively *amplified regions" (class *k *= 7) was associated with a statistical significant high risk of relapse (p < 0.05). In contrast, the proportion of amplifications in regions prone to random chromosomal event was not significantly predictive of outcome. In Figure 4, we plotted the Kaplan-Meier curves when dichotomizing into high score (above the third quartile) versus low score (below the third quartile) computed over the "*exclusively *amplified regions" (chi-square statistic = 7.4, p = 0.006).

The same analysis was conducted for copy loss. We found no statistically significant difference for the score computed over "*exclusively *deleted regions" (class *k *= 3).

## Discussion

In contrast to leukemia, lymphoma and sarcoma where a specific cytogenetic abnormality is usually present, epithelial malignant tumors such as lung adenocarcinomas are often characterized by aneuploidy (complex and multiple chromosome aberrations) which may reflect an alternative form of genetic instability called chromosomal instability [16]. Chromosomal instability leads to numerical and structural abnormalities that are observed at the gross chromosomal level rather than the nucleotide level. Balanced translocations are rare and the observed chromosomal instability leads to imbalanced aberrations in most cases (gain or loss of genetic material). Genomic gains lead to over-expression of oncogenes whereas genomic losses lead to under-expression of tumor-suppressor genes, both resulting in a selective advantage of the cancer cell. The sequential acquisition of genetic alterations occur in individual cell within a population and leads to a wave of clonal expansion due to the relative growth advantage that the new alteration confers to the cell.

When analyzing aCGH experiments on multiple samples of patients, the challenge is to distinguish CNAs that are likely to represent non-random chromosomal events and are thought to involve the critical genes (drivers) from those which are randomly altered during pathogenesis. Given the vast amount of data obtained from high resolution aCGH, biostatistical modeling is required for the discovery of novel regions with propensity for non-random chromosomal events.

In this work, we consider a latent class model-based approach for capturing chromosomal aberration patterns taking into account the interdependencies among propensity of alterations. The primary data processed by our model are the number of deletions and amplifications in each sample that are obtained from a pre-processing of the aCGH signals. A number of algorithms are available to do this. Here, we chose to use CGHmix [6] to label the clones as it has the benefit of taking into account spatial dependencies along the chromosome. Our latent class model is applicable to any preprocessing of the data, of course its output will depend on the initial classification data for each clone in each patient.

In our dataset, we favor the nine-component model but several competing models are tenable ranging from six to nine components. In practice, we think that finding the "best" fit to the data is not the main interest but rather to obtain a good balance between a reasonable fit and sufficient flexibility for describing finely the different levels of genomic aberrations across the whole dataset. This is why we propose the nine-component model as a prime candidate to be estimated if the samples are sufficiently informative.

Considering the present series of stage IB lung adenocarcinomas, our results show that most of the oncogenes and tumor-suppressor genes known to play a role in lung adenocarcinomas are located within exclusively amplified and deleted regions, respectively. This suggests that these latter regions play a substantial functional role in the selective advantage of tumor cells. It is worth noting that this selective process seems to play an important role since about one-third of the genome is classified as exclusively amplified or deleted. Previous studies on various tumors (breast, colorectal, esophageal, endometrioid carcinomas) have shown that an increasing number of chromosomal aberrations correlate with poor prognosis [17]. In our study, we showed that accumulation of amplification occurring within exclusively amplified genomic regions is related with relapse-free survival whereas genomic clones prone to random chromosomal aberrations blurred the impact of copy gains on survival. This result emphasizes that all copy gains may not be equivalently linked to the disease process, and that a subset of clones associated with contrasting patterns between gains and losses over tumor samples could be a more relevant entity. Thus averaging copy gains within a tumor may be too coarse a measure.

As seen from the data, the strong interdependencies between copy loss and copy gain clearly justifies our joint modeling as compared to simple marginal approach with or without permutation procedures. In particular, our approach avoids having to define an arbitrary cutoff for the marginal frequency across the samples and shows that this latter may depend on the chromosomal aberration studied (loss/gain copy).

Constructing background distributions from marginal approaches for deletion and amplification, as is commonly done rather than considering the joint distribution (multinomial) could be misleading when these events are not independents. As an example, when considering the marginal rate of copy loss, the observed deletion rates for the two distinct genomic areas that harbor HDAC4 gene (histone deacetylase, chromosome 2q37) and PDZRN4 (PDZ domain containing zinc finger 4, chromosome 12q12) are the same (16.9%). However, the observed marginal amplification rates are clearly different with 30.5% and 7.7% for HDAC4 and PDZRN4 areas, respectively, advocating the need to consider two different chromosomal patterns for these genomic areas. In our model, these two genomic areas are classified in two different classes: HDAC4 area is listed in class *k *= 8 (complex pattern with high level for both amplification and deletion) whereas PDZRN4 area is listed in class *k *= 2 (background aberration rate). In this case, analyzing marginal deletion rates leads implicitly to define a hybrid state such as the 'non-deletion state' for the null hypothesis which is highly depending on the copy gain state. Our strategy, which is a modeling rather than a hypothesis testing approach, helps to solve this problem by considering copy losses and gains through our multinomial mixture model.

Our method is well suited for an explicit dissection of the complex null hypothesis model. Here, it leads to distinguish between regions with medium levels of loss/gain copy that can be considered as random chromosomal events (background) and regions with refractory patterns. In future studies, we think that investigating these latter regions should be pursued more thoroughly since these may harbor critical region of the genome that are highly resistant to chromosomal instability. With such complex null-hypothesis, computing adjusted p-value from resampling-based method is not straightforward and crucially depends on the null hypothesis model.

Our method leads to prioritize genomic areas prone to non-random chromosomal aberrations but finding driver genes require functional studies. In this setting, it is worth to correlate copy number changes from exclusively amplified/deleted regions to gene expression changes in order to prioritize those that are functionally involved in the tumor process.

## Conclusion

We proposed to identify patterns of chromosomal aberrations across tumor samples from high-resolution comparative genomic hybridization microarrays by modeling copy number states as a multinomial distribution with probabilities parametrized through a latent class model. This model allows distinguishing genomic regions prone to non-random chromosomal aberrations with potential impact on clinical outcome from those prone to random chromosomal aberrations. In a homogeneous series of lung adenocarcinomas, we show that most of the known oncogenes or tumor suppressor genes associated with this tumor type are located within regions with exclusive propensity for either copy loss or copy gain. We also highlight new genomic areas of potential interest and show that an increase of the frequency of amplification in these particular genomic areas is significantly associated with poorer survival. These results suggest that new insights on chromosomal changes may emerge from our modeling approach.

## Abbreviations

CNA: copy number alteration; aCGH: array comparative genomic hybridization; MCMV: Monte Carlo Markov chain.

## Competing interests

The authors declare that they have no competing interests.

## Authors' contributions

All authors read and approved the manuscript. PB: Initial conception and model development, data analysis, manuscript preparation; PT: aCGH supervision, discussion; MA: Responsible for the patients and outcome of patients. SCB: selection, pathological review & validation, manuscript preparation; SR: critical discussion about modeling, data analysis and manuscript preparation.

## Pre-publication history

The pre-publication history for this paper can be accessed here:


